# The Complete Mitogenome of the Wood-Feeding Cockroach *Cryptocercus meridianus* (Blattodea: Cryptocercidae) and Its Phylogenetic Relationship among Cockroach Families

**DOI:** 10.3390/ijms18112397

**Published:** 2017-11-12

**Authors:** Weijun Li, Zongqing Wang, Yanli Che

**Affiliations:** College of Plant Protection, Southwest University, Beibei, Chongqing 400716, China; liweijun201608@163.com (W.L.); zqwang2006@126.com (Z.W.)

**Keywords:** *Cryptocercus meridianus*, Blattodea, mitogenome, gene, rearrangement, phylogenetic analysis

## Abstract

In this study, the complete mitochondrial genome of *Cryptocercus meridianus* was sequenced. The circular mitochondrial genome is 15,322 bp in size and contains 13 protein-coding genes, two ribosomal RNA genes (12S rRNA and 16S rRNA), 22 transfer RNA genes, and one D-loop region. We compare the mitogenome of *C. meridianus* with that of *C. relictus* and *C. kyebangensis*. The base composition of the whole genome was 45.20%, 9.74%, 16.06%, and 29.00% for A, G, C, and T, respectively; it shows a high AT content (74.2%), similar to the mitogenomes of *C. relictus* and *C. kyebangensis*. The protein-coding genes are initiated with typical mitochondrial start codons except for *cox1* with TTG. The gene order of the *C. meridianus* mitogenome differs from the typical insect pattern for the translocation of *tRNA-Ser^AGN^*, while the mitogenomes of the other two *Cryptocercus* species, *C. relictus* and *C. kyebangensis*, are consistent with the typical insect pattern. There are two very long non-coding intergenic regions lying on both sides of the rearranged gene *tRNA-Ser^AGN^*. The phylogenetic relationships were constructed based on the nucleotide sequence of 13 protein-coding genes and two ribosomal RNA genes. The mitogenome of *C. meridianus* is the first representative of the order Blattodea that demonstrates rearrangement, and it will contribute to the further study of the phylogeny and evolution of the genus *Cryptocercus* and related taxa.

## 1. Introduction

*Cryptocercus* is a genus of woodroaches which occur in the high mountainous forests of temperate regions, specifically in the Nearctic, Palaearctic, and Oriental Regions, and feed on rotten wood in logs [1,2,3]. Their distributional pattern has been strongly affected by palaeogeographic events that influenced their source tree hosts, such as the appearance of land bridges and the uplift of mountains [4]. Additionally, *Cryptocercus* is regarded as a link between termites and Blattodea. Recent studies of phylogenetic relationships among Dictyoptera or Blattodea show that *Cryptocercus* is the sister group of termites [1,5,6,7,8]. Therefore this genus is an important model for elucidating the early stages of social evolution in termite eusociality [9].

Insect mitochondrial genomes, which tend to evolve faster than nuclear genes, are very useful for studies of evolutionary genomics and are widely used to discern the phylogenetic relationships at various taxonomic levels and the investigation of population structures [10,11]. Jeon & Park (2015) investigated the mitogenome of *Cryptocercus kyebangensis* and compared it with *Cryptocercus relictus* [12]. They found that *C. kyebangensis* has a close relationship with *C. relictus* and these two *Cryptocercus* species share the same mitochondrial gene order.

*Cryptocercus meridianus* is distributed in Lijiang, Yunnan Province, which is located in the Hengduan Mountains [13]. Che et al. (2016) indicated that *C. meridianus* forms a sister taxon relationship with the group of *C. relictus* from Northeast China and *C. kyebangensis* from Korea, but had a distant phylogenetic relationship with species in the Hengduan Mountains [1]. Given this situation, we sequenced the complete mitochondrial genome of *C. meridianus* and compared it with previously published mitogenomes of *C. relictus* (GenBank: JX144941) and *C. kyebangensis* (GenBank: KP872847). We compared the gene orders in these three members of the *Cryptocercus* genus and found a gene rearrangement in the mitogenome of *C. meridianus*. For phylogenetic analyses, we constructed phylogenetic trees based on 13 protein-coding genes and two rRNAs. In order to discern more accurate phylogenetic relationships and reduce systematic errors from compositional and mutational biases in insect mitochondrial genomes, we used a site-heterogeneous mixture model for Bayesian Inference. In addition, we removed the third codons of the 13 protein-coding genes as they are fast-evolving sites. Despite many studies that have attempted to clarify the phylogenetic analyses within Blattodea based on mitogenomes, the phylogenetic relationships are still unresolved. This is mainly because the mitogenomes of members of the order Blattodea are poorly published. Therefore, more mitogenomes of species in the order Blattodea are needed to be added in ongoing research to establish the phylogenetic relationships.

## 2. Results and Discussion

### 2.1. Mitogenome Organization and Nucleotide Composition

**Figure 1:**
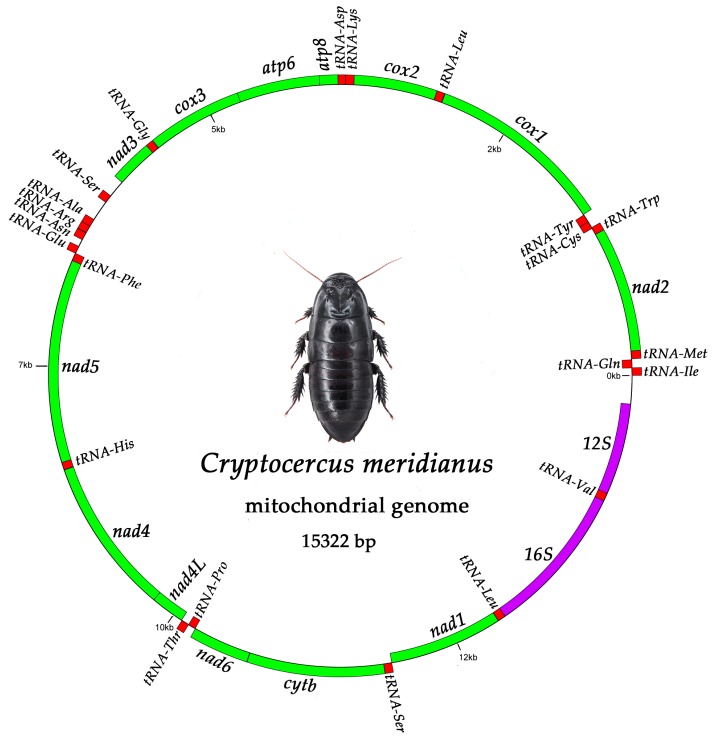
Genetic map of the complete mitochondrial genome of *C. meridianus*. The genes are abbreviated as follows: *nad1*, *2*, *3*, *4*, *4L*, *5*, *6* refer to nicotinamide adenine dinucleotide subunit; *cox1*, *2*, *3* refer to cytochrome oxidase subunit; *16S* refers to large subunit of ribosomal RNA gene, *12S* refers to small subunit of ribosomal RNA gene; *atp6*, *8* refer to ATP synthase F0 subunit; *cytb* refers to cytochrome B. tRNAs are red colored, protein coding genes (PCGs) are green colored, rRNAs are purple colored. Genes on the light strand are encoded in clockwise orientation; genes on the heavy strand are encoded in anti-clockwise orientation.

The complete mitochondrial genome of *C. meridianus* is found to be 15,322 bp in size, which is smaller than that of *C. relictus* (15,373 bp) and *C. kyebangensis* (15,720 bp), with an asymmetric nucleotide composition (45.20% A, 16.06% C, 9.74% G, and 29.00% T) and an AT bias (74.2%) (Table 1). The circular map of the *C. meridianus* mitogenome is shown in Figure 1, drawn by the software GenomeVx [14]. AT content of the *C. meridianus* mitogenome (74.2%) is slightly higher than that of *C. relictus* (73.5%) and slightly lower than in *C. kyebangensis* (74.4%). Similar to other insects, it is a circular double-stranded molecule, which contains a typical set of 13 protein-coding genes (PCGs), 22 transfer RNA genes (tRNAs), two ribosomal RNA genes (rRNAs) and a large non-coding region (A + T rich region or D-loop region) [15,16]. For the whole mitochondrial genome of *C. meridianus*, four PCGs (*nad5*, *nad4*, *nad4L* and *nad1*), eight tRNAs (*tRNA-Gln*, *-Cys*, *-Tyr*, *-Phe*, *-His*, *-Pro*, *-Leu^CUN^*, and *-Val*) and two rRNAs (*12S* and *16S*) are encoded on the light strand, while all the other genes are on the heavy strand [17]. In comparison with *C. relictus* and *C. kyebangensis*, the location of *tRNA-Ser^AGN^* is quite different in the mitochondrial genome of *C. meridianus*, where it is located between *nad3* and *tRNA-Ala*, while in the other two species the *tRNA-Ser^AGN^* gene is located between *tRNA-Asn* and *tRNA-Glu*. The annotation of the whole mitogenome is shown in Table 2. In addition, there are three unusual non-coding sequences inserted along with the mitochondrial gene rearrangement.

AT and GC skews of the mitochondrial gene regions are approximately the same among the three *Cryptocercus* species, but the D-loop regions seem to be an exception. In the D-loop region, the AT skew value of *C. meridianus* (0.37) is much higher than that of *C. relictus* (0.15) and *C. kyebangensis* (0.27), and the GC skew value of *C. meridianus* (−0.52) is much weaker than that of *C. relictus* (−0.09) and *C. kyebangensis* (−0.33) (Table 3).f

### 2.2. PCGs (Protein-Coding Genes)

The total size of the 13 mitochondrial protein-coding genes (PCGs) of *C. meridianus* is 11,190 bp, which can be translated into 3720 amino acid residues, with the exception of stop codons (30 bp). Compared to *C. kyebangensis* and *C. relictus*, the total number of the *C. meridianus* mitochondrial amino acid residues is two more than *C. kyebangensis* and *C. relictus*. Further analysis shows differences in the number of mitochondrial amino acid residues that were found in *nad3* and *nad1*; the *C. meridianus* mitogenome encodes one amino acid residue more than *C. kyebangensis* and *C. relictus* in *nad3* and one amino acid residue less than them in *nad1*.

There are four kinds of start codons (ATG, ATA, ATT, and TTG) included in the mitogenome of *C. meridianus.* The *nad5* gene is initiated with the GTG codon in the mitochondrial genome of *C. kyebangensis* while it is ATG in *C. meridianus* and *C. relictus*. Generally, there are three kinds of stop codons (TAA, TA*, T**) for translation termination. The *C. meridianus* mitogenome is no exception; for the incomplete stop codons, the missed nucleotides may result from post-transcriptional polyadenylation [18]. As the TAA stop codon is completed by the addition of 3′ A residues to the mRNA, the PCG *nad3* harbors the incomplete stop codon TA* in the mitochondrial genome of *C. relictus* while TAA occurs in the other two *Cryptocercus* species. The other PCGs except *nad3* use the same stop codons among the mitochondrial genomes of three *Cryptocercus* species.

**Figure 2:**
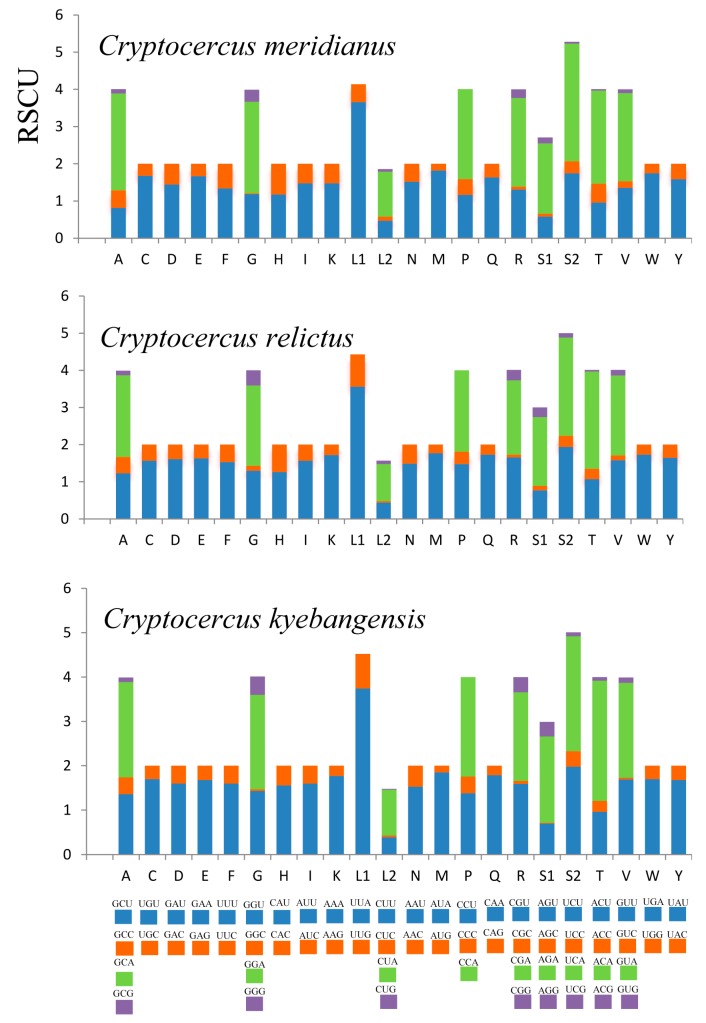
Relative synonymous codon usage (RSCU) for protein coding genes of the three *Cryptocercus* mitochondrial genomes. Codon families are provided on the *x*-axis.

Relative synonymous codon usage (RSCU) was analyzed in the mitochondrial genomes of the three *Cryptocercus* species (Figure 2). In the mitochondrial protein-coding genes of the three *Cryptocercus* species, Ser^UCN^ (S2) was most commonly used in all three *Cryptocercus* species. Furthermore, analysis of the RSCU values for the 13 protein-coding genes indicated an A + T bias [19]. As for protein-coding genes, the A + T bias can be responsible for the frequent use of NNA and NNU (N repesents A, T, C, G) codons. The A + T content of *C. kyebangensis* was slightly higher than in the other two, and the usage of NNA and NNU codons of *C. kyebangensis* was also higher than in the other two *Cryptocercus* species.

**Figure 3:**
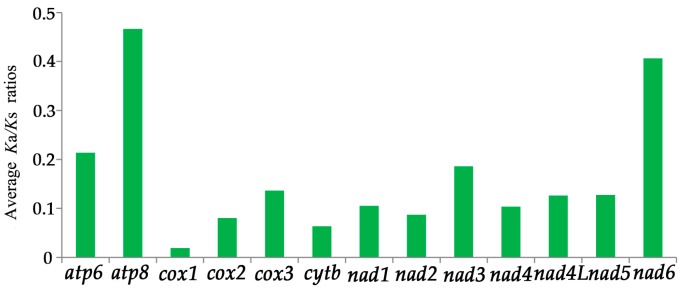
Average *K*a/*K*s ratios of 13 protein-coding genes. *K*a/*K*s is the ratio of non-synonymous substitutions rate (*K*a) to synonymous substitutions rate (*K*s).

The average *K*a/*K*s ratios were calculated for each PCG of the three *Cryptocercus* mitogenomes (Figure 3). It shows that *atp8* has the highest evolutionary rate, followed by *nad6*. *cox1* had the lowest.

### 2.3. The tRNA Genes and rRNA Genes

**Figure 4:**
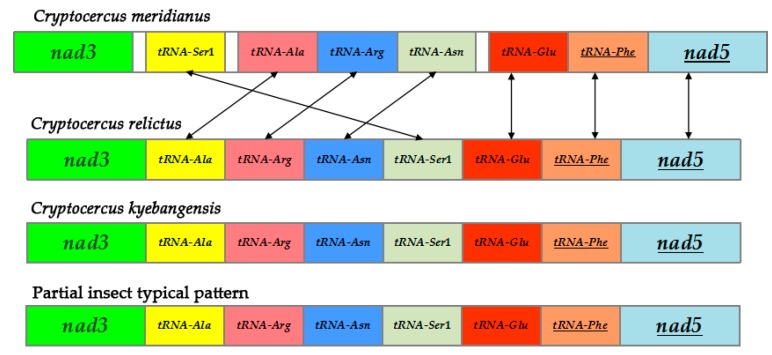
Partial mitochondrial gene arrangements of the three *Cryptocercus* species along with insect typical pattern. The translocation of *tRNA-Ser*1 caused the mitochondrial gene rearrangement of *C. meridianus*, *tRNA-Ser*1 refers to *tRNA-Ser^AGN^*. Genes are not drawn to scale but are arranged practically. Three blank areas refer to unusually long intergenic spacer regions which only occur in the mitogenome of *C. meridianus*. Genes underlined are encoded on the light strand.

The 22 tRNA genes range from 62 to 70 bp in size, and the total length of the 22 *C. meridianus* mitogenome tRNA genes was 1450 bp, which is the same as in *C. relictus* and only one-base different from *C. kyebangensis*. The AT content of the total *C. meridianus* mitogenome transfer RNA genes is 74.9%, which is higher than in *C. relictus* (74.4%) and *C. kyebangensis* (74.6%) (Table 2). Most tRNA sequences can fold into the typical cloverleaf secondary structure [20]. The translocation of *tRNA-Ser^AGN^* is shown in Figure 4. Furthermore, *tRNA-Ser* and *tRNA-Leu* are in double copies, while all the others only have one single copy.

The two rRNA genes of *C. meridianus*’ mitogenome are 777 bp (*12S*) and 1283 bp (*16S*) in size, respectively. 

### 2.4. The D-Loop Region

The D-loop region is located between *tRNA-Ile* and the *12S* gene, which includes the origin sites for transcription and replication [21] and is the control region of the mitochondrial genome. It is found to be 240 bp in size. This region is often the main source of variation in genome length [22], as the non-coding sequences in the D-loop are more prone to mutation [23]. There exists a divergence among the D-loop region sizes in the three *Cryptocercus* species. The D-loop region size of *C. meridianus* is much smaller than that of *C. relictus* (672 bp) and *C. kyebangensis* (1009 bp), so it can be inferred that the mutation rate of the D-loop region in the mitochondrial genome of *Cryptocercus meridianus* is lower than in the other two *Cryptocercus* species, as the mutational region of the former is much shorter than the latter two. In the three *Cryptocercus* species, the AT content of the D-loop region is higher than that of other genes (Table 2): 77.5% in *C. meridianus* D-loop region, which is lower than that of *C. relictus* (80.3%) and *C. kyebangensis* (80.2%). In addition, we also found a 31 bp relatively conserved sequence in the D-loop regions of all three *Cryptocercus* species (Figure S1).

### 2.5. Intergenic Spacer and Overlapping Regions

The total length of *C. meridianus* mitogenome intergenic spacer regions is 414 bp [24], which is much larger than that of *C. relictus* (32 bp) and *C. kyebangensis* (31 bp). There are three long intergenic spacer regions, which are also called non-coding regions. One is located between *tRNA-Ser^AGN^* and *tRNA-Ala* (174 bp); another is located between *nad3* and *tRNA-Ser^AGN^* (147 bp); and the third is located between *tRNA-Asn* and *tRNA-Glu* (62 bp). These three long spacer regions contribute to the unusually large whole intergenic spacer region of the *C. meridianus* mitochondrial genome. In addition, the AT content of these three intergenic spacer regions is higher than in other mitogenome genes. There are seven intergenic overlapping regions ranging from 1 to 8 bp in length, fewer than in *C. relictus* and *C. kyebangensis*, with the latter two having eight intergenic overlapping regions ranging from 1 to 8 bp. The longest overlapping region is consistently located between *tRNA-Trp* and *tRNA-Cys* in the three species.

### 2.6. Rearrangement

Generally, animal mitochondrial genomes have relatively conserved gene arrangements and stable gene content. Although their mitochondrial sequences evolve rapidly over long periods of evolution, their gene arrangements often remain unchanged [25]. With more and more sequences of mitochondrial DNA molecules of insect species being determined [26], the issue of rearrangement of insect mitochondrial genomes has become a focus of more research.

**Figure 5:**
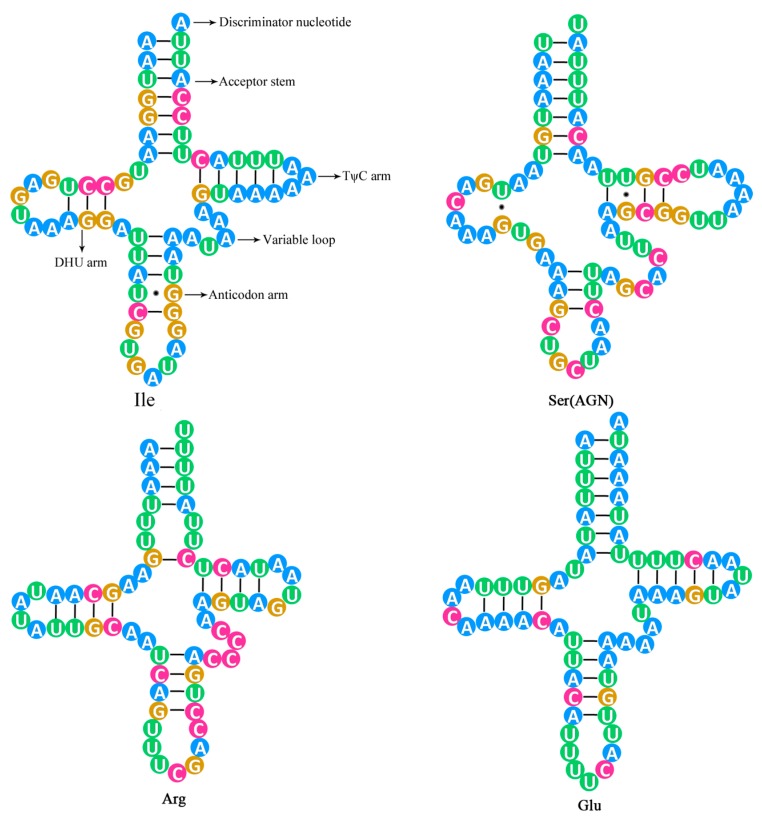
Inferred secondary structure of several tRNA genes for *C. meridianus*.

So far, mitochondrial gene rearrangement has been reported as occurring in many insect species, but Blattodea seems to be an exception. *C. meridianus* is the first species where mitochondrial gene rearrangement has been found in the Blattodea, due to a translocation of *tRNA-Ser^AGN^*. The mitochondrial gene arrangement of the model organism, *Drosophila*, was considered to be the insect pattern formula; while *C. relictus* and *C. kyebangensis* follow this rule, *C. meridianus* does not. The location of *tRNA-Ser^AGN^* is between the *nad3* and *tRNA-Ala* genes in the mitochondrial genome of *C. meridianus*, but in the other two *Cryptocercus* species mitogenome *tRNA-Ser^AGN^* genes are located between the *tRNA-Asn* and *tRNA-Glu* genes. The secondary structure of *tRNA-Ser^AGN^* gene of *C. meridianus* is shown in Figure 5, and the secondary structure of 22 tRNA genes can be seen in Figure S2. Compared with other tRNA genes of *C. meridianus*, the Anticodon arm of *tRNA-Ser^AGN^* is shorter, the Variable loop is longer and it lacks DHU arm. These features contribute to the unstable structure of *tRNA-Ser^AGN^*. 

### 2.7. Phylogenetic Analyses

For the concatenated datasets (PCGR, PCG12R), phylogenetic analyses yielded essentially identical topologies with relatively high support values except for the inner relationship of the group Blaberidae across the topologies for standard Maximum likelihood (ML) and Bayesian inference (BI) analysis using site-homogenous models and Bayesian (PhyloBayes) analyses under site-heterogeneous models. Three recognized major lineages of Blattodea from all inferences were: Corydioidea, Blattoidea, and Blaberoidea, with high support values. AliGROOVE found slight heterogeneity in sequence divergence for a subset of 32 taxa (Figure S3).

**Figure 6:**
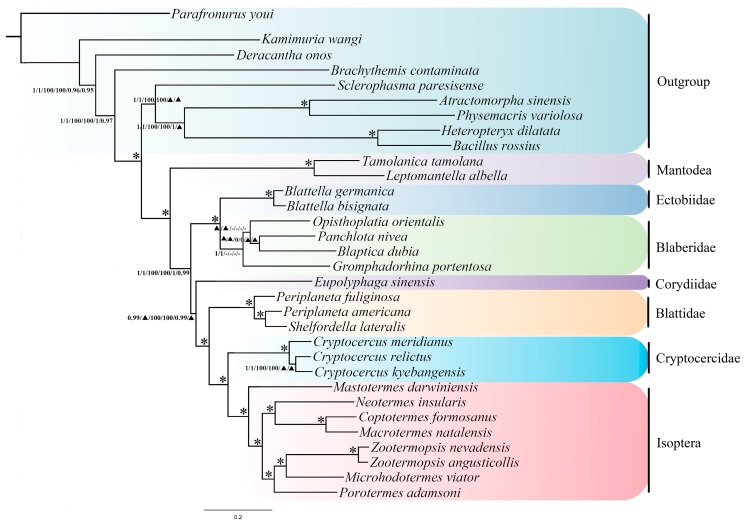
Phylogenetic relationships of major lineages within Blattodea and termites inferred from the concatenated protein-coding genes and ribosomal RNA genes, with the third codon position included (PCGR) and excluded (PCG12R). Branch labels are support for the six analyses in the following order: Bayesian posterior probabilities (BPP) of the Bayesian trees inferred from the dataset PCGR and PCG12R using site-homogeneous models; Bootstrap supports of the maximum-likelihood trees inferred from the dataset PCGR and PCG12R; and Bayesian posterior probabilities (BPP) of the Bayesian trees inferred from the dataset PCGR and PCG12R using site-heterogeneous mixture models. (*) indicates 100% support value for all six analyses. (-) indicates that the node is absent for the corresponding analysis. (▲) indicates BPP below 0.95.

In this study, the topologies of phylogenetic trees are always stable regardless of ingroup change. All datasets show a monophyletic clade containing Cryptocercidae and Isoptera with Blattidae as the sister group of their formed branch (Figure 6). Blaberidae is the sister group of Ectobiidae. *Cryptocercus relictus* is a sister group of *Cryptocercus kyebangensis. Cryptocercus meridianus* is well supported as the sister group of (*Cryptocercus relictus* + *Cryptocercus kyebangensis*). This work was based on several mitogenomes published to date and presents a stable phylogeny of Cryptocercidae and other related lineages. More complete mitogenomes are needed to better reconstruct phylogeny within Blattodea in the future.

The mitogenome of *Cryptocercus meridianus* shows many common features observed in other *Cryptocercus* species, such as AT bias, truncated stop codons, and codon usage. They have the same genome structure which contains 22 tRNA, 13 protein-coding genes (PCGs) and 2 rRNA. The length variation of D-loop regions is the main cause for the diversity of whole genome size; moreover, as for *C. meridianus*, some extra non-coding regions located in *nad3*~*nad5* also contribute to the genome size variation. 

Generally speaking, mitochondrial gene arrangement is stable within major animal lineages [25], however this is not the case for *Cryptocercus*. *C. meridianus* is the first representative of the order Blattodea that demonstrates mitochondrial gene rearrangement, but as this species is poorly sampled, there may be more cases of mitochondrial gene rearrangement in this group. Most probably, the translocation of *tRNA-Ser^AGN^* led to this rearrangement, potentially enabled by the presence of two unconventionally long intergenic spacers on both sides of the *tRNA-Ser^AGN^* gene; this phenomenon is similar to that found in Hymenoptera [27]. In hymenopteran mitochondrial genomes, overlapping genes rarely are involved in rearrangement, and those rearranged genes usually have intergenic regions on both sides. This kind of rearrangement would be gene shuffling, which means that a rearranged gene moves closely on the same strand while never crossing PCGs nearby. Several mechanisms involving the mitochondrial gene have been put forward [28,29,30,31,32,33]. One of the soundest mechanisms suited to explain this phenomenon is described by the duplication-random loss theory; that is, the slipped-strand mispairing took place first [34] and then gene deletions led to the resulting phenomenon. 

Figure 6 shows the results of the six phylogenetic analyses. These results are consistent with the work of Cheng et al. [20] on the sister groups of (Blaberidae + Ectobiidae), but the internal topologies of Blaberidae are heterogeneous among the six analyses. In this section, only the node of sister group (*Panchlota nivea* + *Blaptica dubia*) are stable in these six analyses, but with relatively low support values; the other two nodes are absent in some analyses. In addition, there exists some differences in the clade of Corydiidae: our work indicates the relationship as (Corydiidae + (Blattidae + (Cryptocercidae + Isoptera))), while Cheng et al. [20] report the relationship as (Corydiidae + (Cryptocercidae + Isoptera) + (Blattidae + (Ectobiidae + Blaberidae))). These conflicts may be explained by the choice of taxa used to construct phylogenetic trees, as this result may be due to the lack of key taxon. Currently, published mitogenomes of Blattodea are very scarce, so more mitogenomes of members of the Blattodea should be sequenced to better elucidate these phylogenetic relationships.

## 3. Materials and Methods

### 3.1. Sampling and DNA Extraction

Specimens of *Cryptocercus meridianus* were collected from Lijiang (27.08° N; 100.14° E), Yunnan Province in the Yulong Mountain region of Southwest China. Our study activities were not banned by any organization or individual and did not involve protected or endangered species. Voucher specimens of *C. meridianus* were deposited in the Institute of Entomology, Southwest University (SWU), Chongqing. Specimens of *Cryptocercus meridianus* were preserved in 100% ethanol and stored at −80 °C. Total genomic DNA was extracted from fresh muscle of one leg using the TIANamp Genomic DNA Kit (DP304, TIANGEN, Beijing, China) according to the manufacturer’s protocol.

### 3.2. PCR Amplification, Sequencing and Sequence Assembly

The primers used in this study were referred from [35], which are universal for *Cryptocercus* species. All primers used in this study are provided in Table S1. All reactions were carried out in volumes of 25 µL, containing 14.25 µL of ultrapure water, 2.5 µL of 10× buffer (Mg^2+^ Free), 2 µL of MgCl_2_ (25 mM), 2 µL of dNTP mixture, 1 µL of each primer, 0.25 µL of Taq polymerase, and 2 µL of DNA template. The concentration of Taq stock solutions is 5 units/µL, the primers and dNTPs are 0.5 and 200 µM respectively. The following steps were performed on a programmable thermal cycler. The amplification protocol settings used are: 94 °C for 5 min; followed by 35 cycle 94 °C for 45 s, 48 °C for 45 s, and 72 °C for 45 s; and final extension at 72 °C for 10 min. PCR products were examined by electrophoresis on a 1% agarose gel to confirm PCR availability, and then sequenced via primer walking by BGI Tech (Beijing, China). In addition, the complete mitochondrial genome of *C. meridianus* was also sent for high-throughput sequencing with the Illumina Hiseq 2500 platform by Personal Biotechnology Company, Shanghai, China. Overlapping nucleotide sequences were assembled using SeqMan (DNAStar) and mitoMaker software (http://sourceforge.net/projects/mitomaker/).

### 3.3. Sequence Analysis

The complete mitogenome of *Cryptocercus meridianus* was annotated on the Mitos Web Server (http://mitos.bioinf.uni-leipzig.de/index.py). Then 22 transfer RNA genes were identified by tRNA scan-SE Search Server V.1.21 [36] and ARWEN (online version) [37]. The protein-coding and rRNA genes were inferred based on alignment with other two *Cryptocercus* mitogenomes and BLAST 2.6.0 searches against the GenBank database (https://blast.ncbi.nlm.nih.gov/Blast.cgi). The base composition and relative synonymous codon usage (RSCU) were calculated using MEGA 5 [38]. The average values of *K*a/*K*s across the three *Cryptocercus* species’ pairwise comparisons were calculated by DNASP v.5.0 with Genetic code = mtDNA *Drosophila* [39]. The overlapping regions and intergenic spacers between genes were counted manually as in [40]. AT/GC skew analyses were carried out respectively with the formulas AT skew = [A − T]/[A + T] and GC skew = [G − C]/[G + C] [41].

### 3.4. Phylogenetic Analysis

To infer the phylogenetic relationships within Dictyoptera, the newly generated mitogenomes and previously reported mitogenome sequences from families of cockroaches and other orders were used to reconstruct phylogenetic trees (Table 4). The phylogenetic tree was rooted using *Parafronurus youi* (Insecta: Ephemeroptera) [42], which is shown to have the farmost genetic relationship with other lineages. Each protein-coding gene was aligned individually based on codon-based multiple alignments by using the MAFFT algorithm implemented in TranslatorX with the L-INS-i strategy [43]. Two rRNA genes were individually aligned using the MAFFT 7.0 online server with the G-INS-I strategy [44]. We generated two datasets of each running, (1) PCGR matrix, including all three codon positions of protein-coding genes and two rRNA genes; and (2) PCG12R matrix, excluding the third codon position of protein-coding genes and two rRNA genes. The two datasets were divided by codon positions within each gene, resulting in 41 partitions (first codon positions, second codon positions, third codon positions of 13 protein-coding genes and two rRNAs genes) and 28 partitions (first codon positions, second codon positions of 13 protein-coding genes and two rRNAs genes) respectively. Phylogenetic analyses were performed using maximum likelihood (ML) and Bayesian inference (BI) methods. ML analyses were implemented using RAxML 7.3.0 [45], and BI analyses were implemented using MrBayes 3.2 [46]. For ML analyses, as the software does not allow different substitution models for different partitions, node reliability was estimated using the GTRGAMMA model with 1000 bootstrap replicates. For BI analyses, we determined the best-fit model for each partition with PartitionFinder v1.1.1 [47] (more details in Table S2). Two independent sets of Markov chains were run simultaneously [48], each with one cold and three heated chains for 10,000,000 generations; trees were sampled every 1000th generation and the first 25% of the generations were discarded as burn-in, the remaining samples were used to construct the consensus tree and Bayesian posterior probabilities (BPP). Convergence was inferred when a standard deviation of split frequencies <0.01 was presented. In our RAxML and MrBayes settings, the clustered partitions are totally based on the PartitionFinder result, and the best partitioning scheme can be seen in Table S2.

The heterogeneity of sequence divergence within different datasets was analyzed using AliGROOVE with the default sliding window size [66], and indels of nucleotide datasets were treated as ambiguity and the BLOSUM62 matrix was used as a default amino acid substitution matrix. The obtained scoring distance between sequences in a dataset is then compared with similarity. Values can vary between −1 if comparisons have full random similarity to +1 for comparisons that have totally non-random similarity. This provides an indirect measure of heterogeneity of a given sequence or clade with respect to the full data set [67].

To reconstruct more reasonable and responsible phylogenetic trees and suppress the systematic errors from base compositional and sequence mutational biases in all mitogenomes used in this study, we used PhyloBayes MPI to analyze the phylogenetic relationships based on the site-heterogeneous mixture models (CAT and CAT + GTR) [68]. Similar to the previous analyses, we analyzed two datasets using PhyloBayes each time we added or reduced insect taxa. In each individual analysis, two independent chains starting from a random tree were run for 20,000 cycles, and trees were sampled at each cycle (for more details, see PhyloBayes manual), the result shows that maxdiff = 0.216667 and meandiff = 0.0109477.

## 4. Conclusions

The mitogenome of *C. meridianus* is the first representative of the order Blattodea that exhibits rearrangement. Since mitochondrial gene rearrangements appear to be unique, the rearrangement of the *C. meridianus* mitochondrial gene would be helpful for our further understanding of the phylogeny and evolution of the genus *Cryptocercus* and related species. The result of phylogenetic study shows a little difference from previous studies based on mitochondrial genomes of species in Blattodea, so more data should be added in order to rebuild the phylogenetic relationships among the Blattodea.

## Figures and Tables

**Table 1:** Size, AT content (%) for *C. meridianus*, *C. relictus*, and *C. kyebangensis* mitogenomes.

Gene	*C. meridianus*		*C. relictus*		*C. kyebangensis*	
	Size (bp)	A + T (%)	Size (bp)	A + T (%)	Size (bp)	A + T (%)
PCGs	11,186	73.3	11,183	72.5	11,184	73.4
tRNAs	1450	74.9	1450	74.4	1451	74.6
rRNAs	2060	76.2	2063	75.8	2067	76.5
D-loop	240	77.5	672	80.3	1009	80.2
Genome	15,322	74.2	15,373	73.5	15,720	74.4

**Table 2:** Annotation of the complete mitogenome of *Cryptocercus meridianus*.

Gene Name	Strand	Location	Size (bp)	IGS	Anticodon	Start/Stop Codon	
*tRNA-Ile*	H	1–65	65		GAT		
*tRNA-Gln*	L	64–132	69	−2	TTG		
*tRNA-Met*	H	140–205	66	7	CAT		
*nad2*	H	206–1233	1028	0		ATG/TA*	
*tRNA-Trp*	H	1234–1299	66	0	TCA		
*tRNA-Cys*	L	1292–1355	64	−8	GCA		
*tRNA-Tyr*	L	1356–1424	69	0	GTA		
*cox1*	H	1429–2964	1536	4		TTG/TAA	
*tRNA-Leu^UUR^*	H	2967–3032	66	2	TAA		
*cox2*	H	3033–3717	685	0		ATA/T**	
*tRNA-Lys*	H	3718–3787	70	0	CTT		
*tRNA-Asp*	H	3788–3849	62	0	GTC		
*atp8*	H	3850–4008	159	0		ATT/TAA	
*atp6*	H	4002–4682	681	−7		ATG/TAA	
*cox3*	H	4682–5470	789	−1		ATG/TAA	
*tRNA-Gly*	H	5475–5538	64	4	TCC		
*nad3*	H	5536–5892	357	−3		ATA/TAA	
*tRNA-Ser^AGN^*	H	6040–6108	69	147	GCT		
*tRNA-Ala*	H	6283–6347	65	174	TGC		
*tRNA-Arg*	H	6348–6410	63	0	TCG		
*tRNA-Asn*	H	6414–6478	65	3	GTT		
*tRNA-Glu*	H	6541–6604	64	62	TTC		
*tRNA-Phe*	L	6605–6671	67	0	GAA		
*nad5*	L	6672–8400	1729	0		ATG/T**	
*tRNA-His*	L	8401–8464	64	0	GTG		
*nad4*	L	8467–9807	1341	2		ATG/TAA	
*nad4L*	L	9801–10,088	288	−7		ATG/TAA	
*tRNA-Thr*	H	10,093–10,157	65	4	TGT		
*tRNA-Pro*	L	10,158–10,221	64	0	TGG		
*nad6*	H	10,224–10,721	498	2		ATT/TAA	
*cytb*	H	10,724–11,855	1132	2		ATG/T**	
*tRNA-Ser^UCN^*	H	11,856–11,923	68	0	TGA		
*nad1*	L	11,920–12,886	967	−4		ATG/T**	
*tRNA-Leu^CUN^*	L	12,888–12,954	67	1	TAG		
*16S*	L	12,955–14,237	1283	0			
*tRNA-Val*	L	14,238–14,305	68	0	TAC		
*12S*	L	14,306–15,082	777	0			
D-loop		15,083–15,322	240	0			

H/L indicates that the gene is encoded on the heavy/light strand, while D-loop region is non-coding. T** or TA* represents incomplete stop codons. Intergenic spacer region (IGS) denotes the length of the intergenic spacer region, for which positive numbers/negative numbers indicate intergenic/overlapping regions between adjacent genes.

**Table 3:** AT skew and GC skew of *C. meridianus*, *C. relictus* and *C. kyebangensis* mitogenomes.

Gene	*C. meridianus*		*C. relictus*		*C. kyebangensis*	
	AT Skew	GC Skew	AT Skew	GC Skew	AT Skew	GC Skew
PCGs	0.22	−0.23	0.24	−0.23	0.23	−0.23
tRNAs	0.12	−0.16	0.15	−0.16	0.14	−0.15
rRNAs	0.29	−0.36	0.30	−0.34	0.31	−0.34
D-loop	0.37	−0.52	0.15	−0.09	0.27	−0.33
Genome	0.22	−0.25	0.23	−0.23	0.24	−0.24

**Table 4:** GenBank accession numbers of taxa used to reconstruct phylogenetic trees.

Order	Species	Accession Number	Reference
Ephemeroptera	*Parafronurus youi*	EU349015	[42]
Odonata	*Brachythemis contaminata*	NC_026305	[49]
Mantophasmatodea	*Sclerophasma paresisense*	NC_007701	[50]
Orthoptera	*Deracantha onos*	EU137664	[51]
	*Physemacris variolosa*	NC_014491	[52]
	*Atractomorpha sinensis*	NC_011824	[53]
Plecoptera	*Kamimuria wangi*	KC894944	[54]
Phasmatodea	*Bacillus rossius*	GU001956	[55]
	*Heteropteryx dilatata*	AB477468	[56]
Mantodea	*Tamolanica tamolana*	NC_007702	[35]
	*Leptomantella albella*	NC_024028	[57]
Isoptera	*Mastotermes darwiniensis*	NC_018120	[35]
	*Microhodotermes viator*	NC_018122	[35]
	*Neotermes insularis*	NC_018124	[35]
	*Zootermopsis nevadensis*	NC_024658	[58]
	*Zootermopsis angusticollis*	NC_018123	[35]
	*Macrotermes natalensis*	NC_025522	[59]
	*Coptotermes formosanus*	AB626147	[60]
	*Porotermes adamsoni*	NC_018121	[35]
Blattodea	*Cryptocercus meridianus*	MG518617	this study
	*Cryptocercus relictus*	NC_018132	[35]
	*Cryptocercus kyebangensis*	KP872847	[12]
	*Blattella germanica*	NC_012901	[61]
	*Eupolyphaga sinensis*	NC_014274	[62]
	*Blattella bisignata*	NC_018549	[63]
	*Periplaneta americana*	NC_016956	[61]
	*Periplaneta fuliginosa*	AB126004	[64]
	*Opisthoplatia orientalis*	KT893460	[65]
	*Panchlota nivea*	KU684412	[20]
	*Shelfordella lateralis*	KU684413	[20]
	*Gromphadorhina*	KU684411	[20]
	*portentosa*		
	*Blaptica dubia*	KU684410	[20]

## Supplementary Materials

Supplementary materials can be found at www.mdpi.com/1422-0067/18/11/2397/s1.

## Abbreviations

*atp6*	ATPase subunit 6
*atp8*	ATPase subunit 8
*cox1-3*	cytochrome c oxidase subunit I-III
*cytb*	cytochrome b
*nad1-6*, *4*L	NADH dehydrogenase subunit 1-6, 4L
PCGs	protein-coding genes
tRNA	transfer RNA genes
rRNA	ribosomal RNA genes
*12/16S*	small and large subunit of ribosomal RNA genes
ML	maximum likelihood analyses
BI	bayesian inference analyses
RSCU	relative synonymous codon usage
IGS	intergenic spacer region
BPP	bayesian posterior probabilities
