# Supplementary material for: The Complete Mitogenome of the Wood-Feeding Cockroach Cryptocercus meridianus (Blattodea: Cryptocercidae) and Its Phylogenetic Relationship among Cockroach Families
Source: Int J Mol Sci. 2017 Nov 12;18(11):2397. doi: 10.3390/ijms18112397 (PMC5713365; doi:10.3390/ijms18112397)
Supplement: Supplementary file 1 [file ijms-18-02397-s001.pdf]

Supplementary:

C. meridianus

TAAGGTAAATAAAAAATCTAAGGAACTACATTTTTAACAACGAAATAAATACAA
AAAAACAAGAAAAATGCAACTAAGATAATCAAATTAATCCCTCTATTAT**AAATA**
AAATTTTTCTTGCCCAACTAAATAGAAAAAACTTTCCAAAAACAAATCCGAAAA
TAAAACTATTCACAAACAAATAAAATTCTAACTTTAATAAACAACCCAACCCGCA
TCAAATAAAGTTTCTATAAATAA

C. relictus

TAAAGCAAATCAAAAATCTCAGGAACAAAATCGTAAACAAAGAAATGAGCAATA
AAAAACAAAAAAAAGCAATTAATAAAACCGAAATTTTTCCATCCAT**AAATAAAA**
TTTTTCTTGCCCAACTAAATAGATAAAACTTTCCAAAAATAAATTCAAAAATAAA
ACTATTCTAAGGTATAAATTAAATTTACGGATCGATCCCTTAATAATAGTTCCATT
GAATATAAGGAGTAATAACACTTTCCTAGATCTCATTTTTTTTTTATTATATAAATG
AGATCAGGAAAGTGTTATTACTCTTTATAGTTTAATATAAGAATTTAAATACATGT
ACCAATTATTGGATCATATTAATTATTATTATATAAGAATTAAATTACAAGATAAT
TATTATTGGTTAGTATAATATAATGTATTTAATTAATAGGTAATACTATTCTATTTA
AATATAAAATGTATTTAAATAAGATTCCATATATTCAACTAAATTCCTTATATATA
TATACTCTATTTTTTTTCTATATAGAGCTATATGATATATAGGTTTAAAGTGTATAA
ACATAAGAATTTATATTAATATGGCAAAAAAAAAATATTATCAACGGACAAACA
AGTTTTAACCAGAAAATAAGTTTCAAGCCAAATAAAAAAAAAGTTCTATAAAATA
ATAAAA

C. kyebangensis

TAAAGTAAAACAAAAATTTCAGGATCACTACCCCAAATAAAGAAACAAATAAAA
AAAAATAAAAAAAAGCAACTAATTAAAAATCAAACTTTCCAGCAC**AAATAAAAT**
TTTTCTTGCCCAACTAAATAGATAAAACTTTTCAAAAATAAAACTAAAAATAAA
ACTATTATAAAGTAAAACAAAAATTTCAGGATCACTACCCCAAATAAAGAAACA
AATAAAAAAAAATAAAAAAAAGCAACTAATTAAAAATCAAACTTTCCAGCACAA
ATAAAATTTTTCTTGCCCAACTAAATAGATAAAACTTTTCAAAAATAAAACTAAA
AATAAAACTATTATAAAGTAAAACAAAAATTTCAGGATCACTACCCCAAATAAA

GAAACAAATAAAAAAAAATAAAAAAAAGCAACTAATTAAAAATCAAACTTTCCA

GCACAAATAAAATTTTTCTTGCCCAACTAAATAGATAAAACTTTTCAAAAATAAA

ACTAAAAATAAAACTATTATAAAGTAAAACAAAAACCCACGGATCAAACCCTTA

ATAATAGTTCCATTGAATATAAGGAGTAATAACACTTTCCTAGATCTCATTTTTTTT

ATTTTATATAAATGAGATCTAGGAAAGTGTTATTACTCTTTATAGTTTACTATAAG

AATTTAAGTTGACATACTCATTATTGGAACATATTTATTATTATAATATAAGAATT

AAATTACAAGATAATTATTATTGGATAGTATAATATAATATATTTATCTAATATGA

ATAATACCATTATATTCATAAATAAAAATATATTTAAATAAGATTCAATATGTTTA

ACTAAATTCCTTATATATGTATAATCTATTTCTCTCTATATAGAGCTATATGATATA

TAAGTTCAAAGTGTATATACATAAGACTTTATATTAATATGGCAAAAAAAAAATA

TTATATAAAGACAAATAAAACATAAACCAAAAAATGAAACCCAAGCCATATCAT

TAAAAAGTTACATAAAATAT

Figure S1. The D-loop regions sequence of the three *Cryptocercus* species' mitochondrial genomes. Bold and underlined segments indicate the 31bp relatively conserved sequence in the three *Cryptocercus* species. Framed segment indicates TA repeats. Dotted segments indicate A/T single copy sequences.

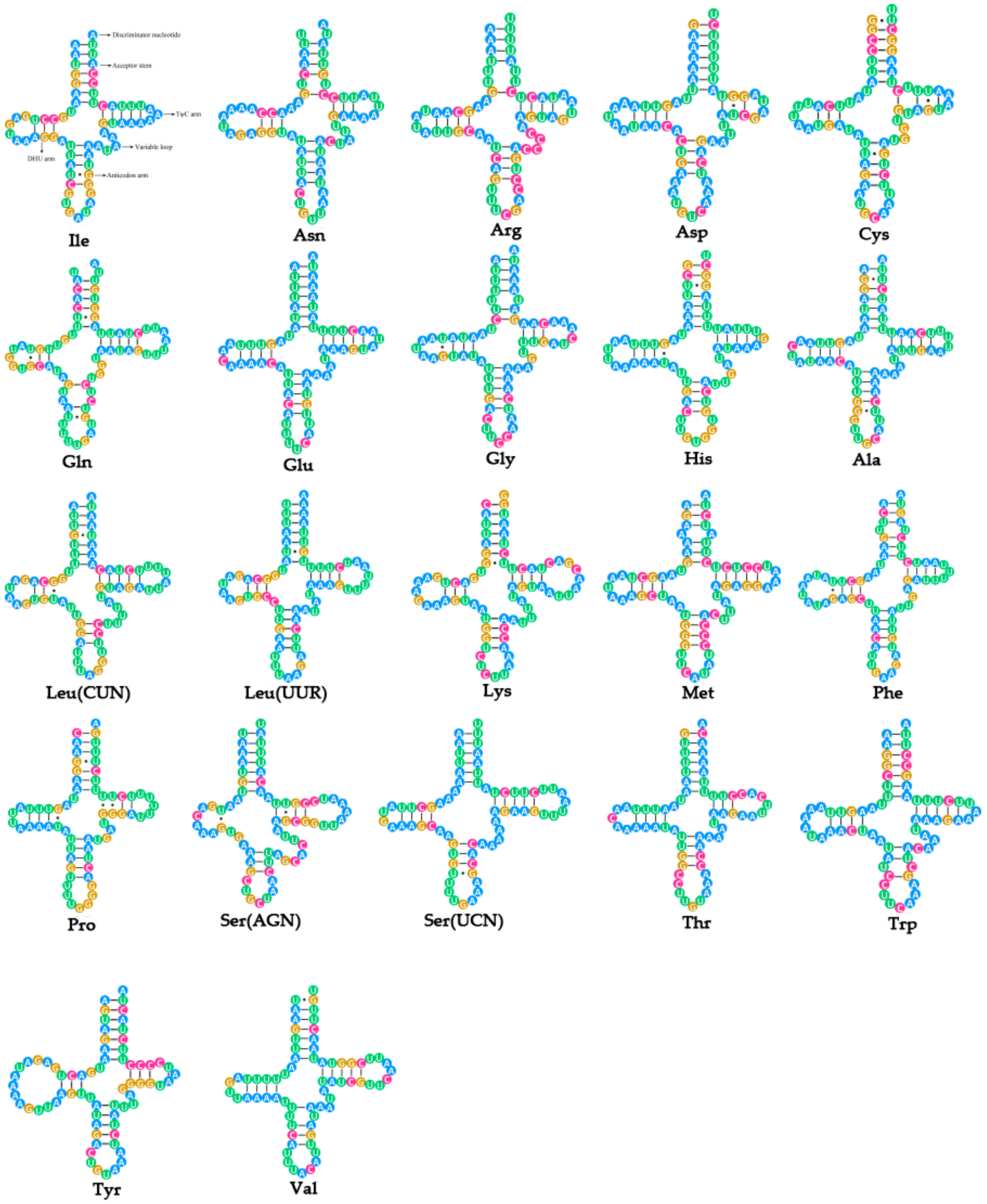

Figure S2. Inferred secondary structure of 22 tRNA genes for *C. meridianus*.

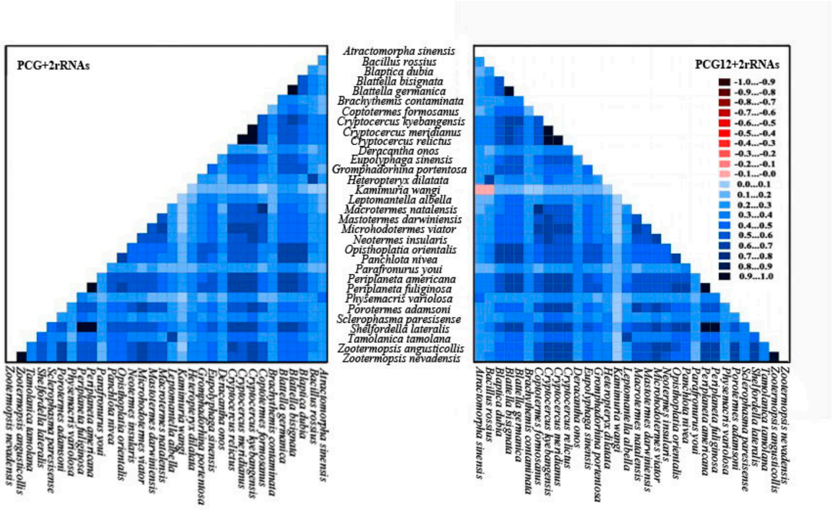

Figure S3. AliGROOVE result of concatenated gene analyses on two datasets. The mean similarity score between sequences is showed by a colored square, based on AliGROOVE scores from -1, indicating a great difference in rates from the other sequences of the data set, that is, heterogeneity (red coloring), to +1 indicating that rates match all other comparisons (blue coloring).

Table S1. Primers used in this study.

Region	Primers (F & R)	Sequence (5′ →3′)
cox1 → *cox2*	C1-J-1718[1]	GGAGGATTTGGA AAT TGATTAGTTCC
	R Lys[3]	GAGACCAGTACTTGCTTTCAGTCATC
cox2 → *nadh4*	CRYs6[2]	AGCAGATGCTACACCAGGACG
	CRYs7[2]	AGATCTTGTAATATAGCCGCTCCC
subPCR *cox2* → *nad5*	CRYs6[2]	AGCAGATGCTACACCAGGACG
	CRYs25[2]	ATTGACTGTTTGTTATTCATTTCG
subPCR *nad5* → *nad4*	CRYs24[2]	TATATCTCAATCTACTGATGAGG
	CRYs13[2]	TCCTTCTTTAGTGCTGTTTATACAC
nad4 → *rrnL*	CRYs8[2]	AGTAGGAATCAA GCTACCCTC
	CRYs2[2]	ACTAAATTACCTTAGGGATAACAGCG
rrnL → *cox1*	CRYs1[2]	ATT ATG CTA CCT TTG CAC GGT C
	CRYs3[2]	ACT AAT CAG TTA CCA AAT CCT CCG

Table S2. The best-fit model for each partition in BI analyse.

Dataset	Best partitioning scheme for Mrbayes (PCGR)		Best partitioning scheme for Mrbayes (PCG12R)	
	**Best model**	**Partitions**	**Best model**	**Partitions**
1	GTR+I+G	12S, 16S, atp6_P1, atp6_P2, atp8_P1, atp8_P2, cox1_P1, cox1_P3, cox2_P1, cox2_P2, cox3_P1, cox3_P2, cytb_P1, cytb_P2, nad2_P1,nad3_P1, nad3_P2, nad4_P1, nad4_P3, nad4L_P2, nad4L_P3, nad5_P2, nad5_P3,nad6_P1	GTR+I+G	12S, 16S, atp6_P1, atp6_P2, atp8_P1, atp8_P2, cox1_P1, cox2_P1, cox2_P2, cox3_P1, cox3_P2, cytb_P1, cytb_P2, nad1_P2, nad2_P1, nad3_P1, nad3_P2, nad5_P1, nad5_P2, nad6_P1
2	HKY+I+G	atp6_P3, atp8_P3, cox3_P3, cytb_P3, nad2_P3, nad3_P3, nad6_P3	TrN+I+G	nad4_P1
3	TVM+I+G	cox1_P2, cox2_P3, nad1_P1, nad2_P2, nad4_P2, nad4L_P1, nad6_P2	TVM+I+G	cox1_P2, nad2_P2, nad6_P2
4	GTR+G	nad1_P2	K81uf+G	nad1_P1, nad4_P2, nad4L_P1, nad4L_P2
5	K81uf+G	nad1_P3		
6	TIM+I+G	nad5_P1		
